# Self-calibrating neuromorphic system for adaptive environmental sensing

**DOI:** 10.3389/frai.2026.1816292

**Published:** 2026-06-02

**Authors:** Anantharaman Prasad, S. Sofana Reka, Prakash Venugopal

**Affiliations:** 1 School of Electronics Engineering, Vellore Institute of Technology, Chennai, India; 2 Centre for Neuroinformatics, School of Electronics Engineering, Vellore Institute of Technology, Chennai, India

**Keywords:** event-driven computation, low-power microcontroller, neuromorphic computing, precision agriculture, soil moisture sensing, spiking neural networks

## Abstract

Precision agriculture demands accurate, real-time environmental monitoring, conventional soil moisture sensors face critical issues such as long-term drift, high energy consumption, and limited adaptability to dynamic environmental changes. These limitations often lead to suboptimal irrigation decisions, wasted resources, and unreliable data, especially in remote or resource-constrained farming regions where frequent manual recalibration is impractical or impossible. This work addresses these challenges by introducing a novel self-calibrating neuromorphic system for adaptive soil moisture sensing. The system leverages Spiking Neural Networks (SNN) deployed on a low-power STM32H563ZI microcontroller. Our proposed solution autonomously recalibrates sensors to mitigate drift, significantly reduces energy consumption through event-driven computation, and adapts seamlessly to changing environmental conditions. The SNN model achieved a Mean Absolute Error (MAE) of 0.4557 and a Root Mean Squared Error (RMSE) of 0.5850, reducing baseline drift from 5.3% to 1.6% over a two-month deployment outperforming models like Isolation Forests and Autoencoders in predictive accuracy. This work significantly contributes to the growing field of neuromorphic computing in IoT applications, offering a scalable, low-power solution for precision agriculture and broader environmental monitoring. The demonstrated effective deployment of SNN-based learning mechanisms on low-constrained microcontroller hardware opens new avenues for resilient, decentralized intelligence in smart homes, wearables, and autonomous infrastructure inspection.

## 1 Introduction

The international farming community is experiencing a technological revolution driven by the simultaneous need for increased food production and diminishing natural resources. Precision farming, which leverages sensor networks, IoT sensors and intelligent systems has become essential for ensuring sustainable and high yield agricultural practices (Getahun et al., 2024). Within this domain, soil moisture is a critical environmental factor that directly impacts irrigation planning, crop health, yields, and water resource management. Accurate and real-time soil moisture monitoring empowers farmers to make informed decisions that optimize productivity and environmental sustainability (Dorigo et al., 2015). Despite continuous advancements in sensor technologies, conventional soil moisture sensing systems are burdened by significant operational challenges. These issues include long-term sensor drift, degradation over time, and non-linear responses influenced by environmental factors such as temperature changes, humidity, and soil heterogeneity (Bogena et al., 2007). Sensor drift, defined as the gradual divergence of sensor output from its true value over time, can lead to critical errors in field deployment, resulting in water wastage or inadequate crop irrigation. A major limitation of most current systems is their reliance on static calibration models. These models are configured at initial deployment and do not adapt to varying environmental or sensor conditions, making them unsuitable for long-term autonomous operation. Furthermore, many agricultural fields are remote or resource-limited, making regular manual recalibration, replacement, or maintenance of sensors impractical or impossible (Kim et al., 2008). This creates a pressing need for the development of self-sustaining, intelligent systems capable of continuous, on-device learning and self-calibration, all while maintaining low power consumption. Traditional deep learning techniques are often unfeasible in these environments due to their high computational expense and power requirements. This research aims to bridge this gap by developing a robust, energy-efficient, and adaptive sensing system that overcomes these inherent limitations. Neuromorphic computing inspired by the structure and efficiency of the human brain, offers a revolutionary paradigm for embedded intelligence (Indiveri and Liu, 2015). Spiking Neural Networks (SNNs), as third-generation neural models, utilize discrete spikes to represent and communicate information, enabling asynchronous and event-based computation that closely mimics biological neurons (Pfeiffer and Pfeil, 2018). Their inherent sparsity and temporal processing capabilities make SNNs particularly well-suited for efficient operation on low-power microcontrollers and edge devices, ideal for real-time environmental sensing applications (Tavanaei et al., 2019). While quantized deep neural networks (DNNs) and TinyML frameworks reduce inference cost, they still rely on continuous-valued activations and dense matrix operations; SNNs, in contrast, communicate through sparse binary spikes, achieving further energy reduction on event-driven activation without sacrificing temporal representational capacity.

In this experimental work, a novel self-calibrating neuromorphic system is introduced specifically for soil moisture sensing on resource-constrained embedded hardware. The system employs an SNN model, trained and deployed on an STM32H5 microcontroller (a low-energy ARM Cortex-M33 microcontroller), to perform real-time inference on temporal soil moisture signals. Unlike systems that rely solely on raw sensor readings, our system continuously compares predicted and observed moisture levels to dynamically update calibration parameters. This adaptive adjustment mechanism compensates for drift by analysing error residuals and adjusting gain and offset parameters in real-time via a lightweight feedback loop (Lane et al., 2016). A key innovation is the integration of a self-calibration loop with neuromorphic inference, enabling reliable system operation without external intervention. As soil conditions change or sensor properties degrade over time, the system automatically detects these changes and recalibrates sensor readings to maintain consistency with the SNN’s internal predictive model. In contrast to conventional filtering or curve-fitting methods, our approach employs online learning from residual patterns to ensure adaptability and long-term reliability. The proposed system also integrates IoT-based communication protocols for wireless data transfer and remote observation, facilitating its integration into smart farming networks. The low-power, low-latency operation of the SNN ensures system functionality even with limited energy budgets, making it ideal for solar or battery-powered agricultural deployments.

The main contributions of this research work are:

- The implementation and deployment of a spiking neural network model for real-time soil moisture estimation on embedded hardware.
- The creation of a lightweight, adaptive self-calibration mechanism that uses SNN prediction residual as a direct calibration signal, enabling on-device drift compensation within a 128 KB memory footprint without external reference sensors.
- Experimental verification of the system in simulated and real-world environments, showing its accuracy, energy efficiency, and long-term robustness.

Therefore, we propose a combined anomaly detection and adaptive recalibration framework that leverages SNNs to achieve reliable, long term autonomous operation without external intervention.

In the remainder of this paper, section 2 provides background information on SNN, IoT-based environmental sensing, and self-calibration mechanisms. Section 3 details the proposed methodology, including the system architecture, data acquisition, SNN model design and training, anomaly detection, adaptive recalibration, and edge hardware integration. Section 4 presents and discusses the experimental results, covering SNN model performance, the impact of calibration, training loss, comparison with traditional models, and deployment on edge hardware. Finally, Section 5 concludes the paper and outlines directions for future work.

## 2 Background

This section provides a brief background of SNN, IoT based environmental sensing, self-calibration mechanism.

### 2.1 Spiking neural networks [SNN]

**Figure 1:**
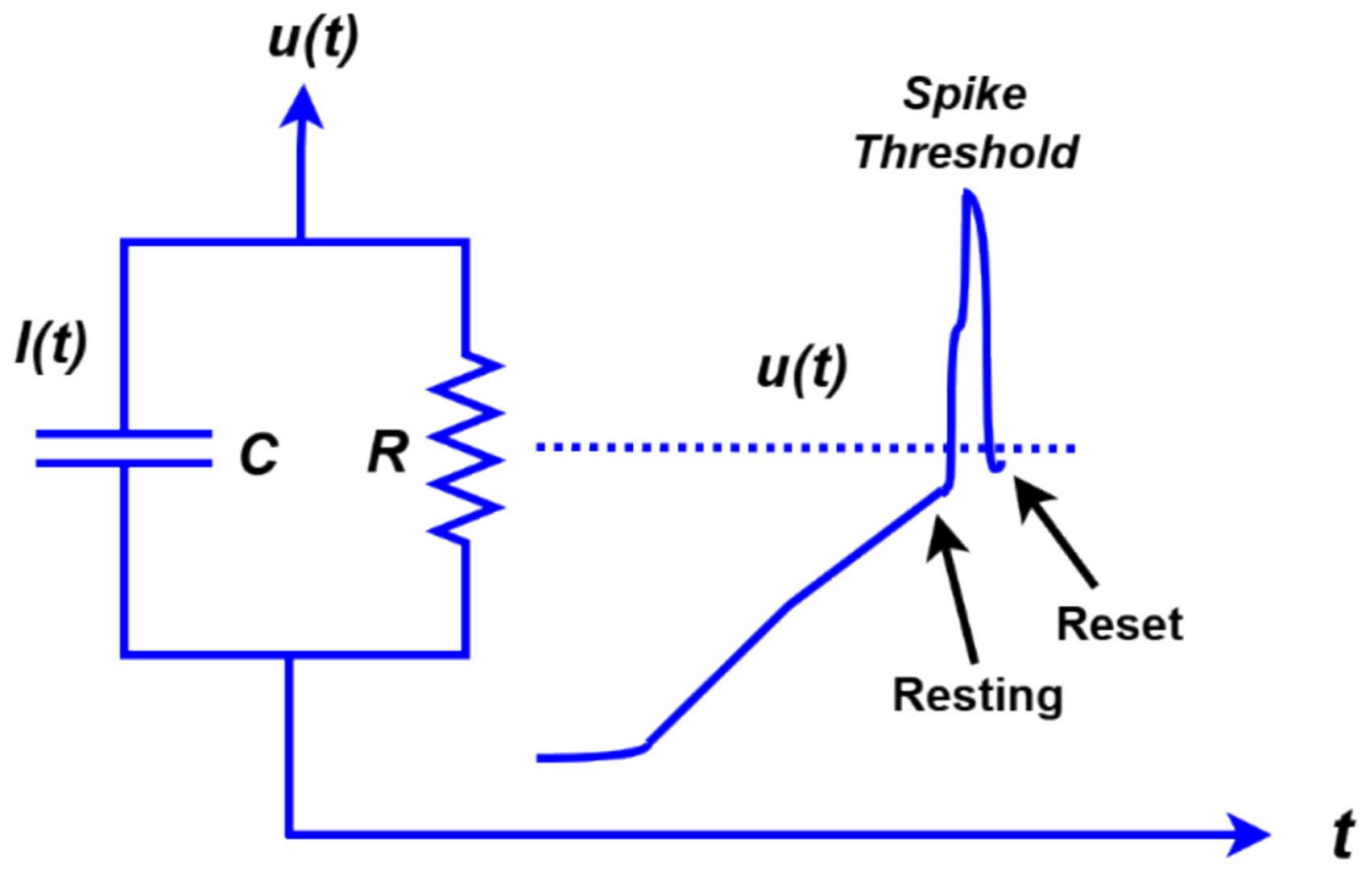
Leaky-integrate-and-fire neuron model.

SNN are the third generation of artificial neural networks, which seek to more accurately capture the event-driven nature of biological neurons than the traditional artificial neural networks [ANNs]. SNNs compute data with discrete spikes, as opposed to traditional deep learning models that compute data with continuous-valued activations (Auge et al., 2021). This allows for efficient temporal and spatial information encoding and processing in a biologically plausible fashion. The simplest building block of SNN is the spiking neuron, which captures the temporal dynamics of the membrane potential of the biological neuron (Nunes et al., 2022). Event-driven nature of SNNs leads to less power consumption, and therefore SNNs are even more appropriate for neuromorphic hardware and edge computing (Wang et al., 2024). The Leaky Integrate-and-Fire [LIF] model, as illustrated in Figure 1, is the simplest and most widespread abstraction of the spiking neuron (Venkatesha et al., 2021; Butcher, 2016). The model captures the dynamics of a biological neuron by considering the dynamics of the membrane potential in terms of *V*(*t*).

To this end, the LIF neuron integrates input synaptic currents in terms of current spikes, and its dynamic activity is characterized by the following differential Equation 1.


$$\tau_{m}\frac{\mathrm{dV}(t)}{\mathrm{dt}}=-(V(t)-V_{\text{reset}})+R_{m}I(t)$$ (1)

Where *V*(*t*) is the membrane potential at time *t*, *V*_reset_ is the resting potential of the neuron, R_m_ is the membrane resistance, *I*(*t*) is the input current received from presynaptic neurons, and $\tau_{m}=R_{m}C_{m}$ is the membrane time constant, where *C_m_* is the membrane capacitance. The right-hand side negative term is the leakage effect that makes the membrane potential decline toward its resting level in the lack of input. The term *V*(*t*) represents the passive discharge of membrane charge toward the resting potential V_reset modelling the ionic leakage current through cell membrane. The product *R_m_I*(*t*) scales the current through cell membrane resistance, converting current to a voltage increment. Together, these terms capture the integrate-and-fire dynamic in a physically interpretable form. This renders the LIF model more biologically plausible than the IF model that is based on perfect integration with no decay.

When the membrane potential reaches a certain threshold *V_th_*, the neuron emits a spike, and the potential is reset to a predefined value *V*_reset_, which is the resting potential as shown in Equation 2.


$$V(t)=\{\begin{array}{c} V_{\text{reset}},\text{if}\;V(t)\geq V_{\mathrm{th}}\\ \;\;V(t),\;\;\text{Otherwise} \end{array}$$ (2)

This relies on how biological neurons produce action potential upon receipt of a size of depolarization. This reset mechanism mirrors the biological absolute refractory period, during which a neuron is temporarily hyperpolarized and cannot fire again immediately, thereby naturally limiting maximum firing rate and introducing temporal sparsity. Solving differential equations by continuous computation is computationally costly and inefficient, particularly in high-dimensional or complicated systems as it entails fine discretization and iterative numerical algorithms (Butcher, 2016). It is therefore typically discretized to the update rule form for functional use in simulation and neuromorphic systems (Mehonic et al., 2020). The discrete time LIF neuron, in which the membrane potential is updated at discrete time steps, is given by Equation 3.


$$V_{i}^{t}=\lambda V_{i}^{t-1}+\Sigma \omega_{\mathrm{ij}}o_{j}^{t}$$ (3)

Where $V_{i}^{t}$ is the potential of neuron *i* at timestep *t*, *λ* is a leakage factor [0 *< λ <* 1] for leak, *ꞷ_ij_* is the synapse weight from presynaptic neuron *j* to neuron *i*, and *o*^*t−*1^ is the previous timestep’s spike output of neuron *j* (Xie et al., 2022). This equation effectively captures the way the neuron sums weighted inputs, compensating for the decay of membrane potential, and so is more computationally tractable than solving the differential form.

A neuron produces a spike when its membrane potential passes the threshold V_th_ which can be written mathematically as Equation 4.


$$o_{i}^{t}=\{\begin{array}{c} 1,\text{if}\;V(t)\geq V_{\mathrm{th}}\\ \;0,\;\;\;\;\text{otherwise} \end{array}$$ (4)

Where $o_{i}^{t}$ is the binary spike output. This step function, however, poses a significant challenge in the training of SNNs using conventional gradient-based techniques since the derivative of a step function is zero everywhere and undefined at the threshold (Rathi et al., 2020).

To solve this issue, surrogate gradient methods use a smooth function to estimate the derivative of the threshold function (Neftci et al., 2019). A very popular approximation is expressed as a piecewise linear function Equation 5.


$$\frac{\partial o_{i}^{t}}{\partial V_{i}^{t}}=\xi \max(0,1-\frac{|V_{i}^{t}-V_{\mathrm{th}}|}{V_{th}})$$ (5)

**Figure 2:**
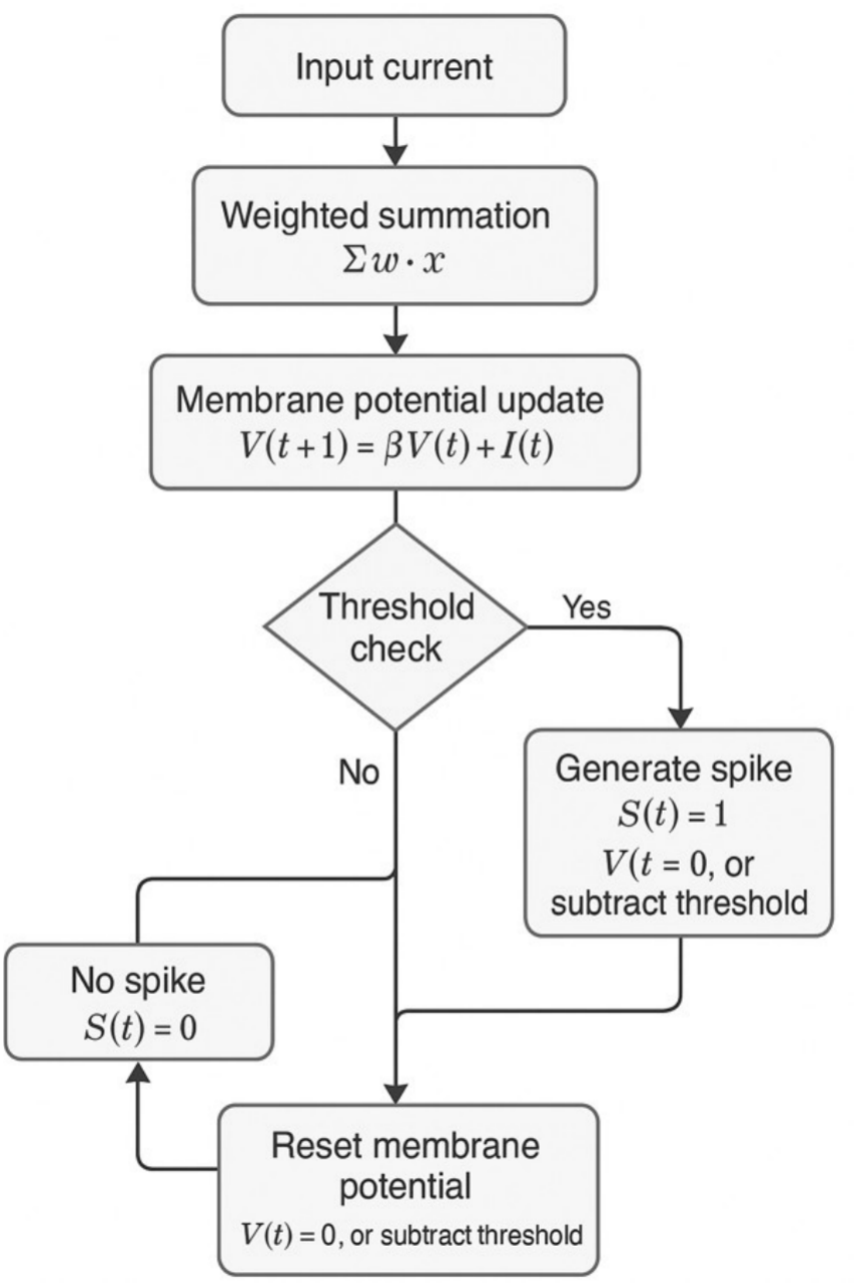
Flowchart of model spiking neuron model.

In which *ξ* is a scaling parameter determining the flow of the gradient which was used in default snnTorch configuration and set to 1. This allows for the application of back propagation-like procedures to train SNNs with spike-based computation maintained. Figure 2 illustrates the step-by-step process of membrane potential update and spike generation in the proposed spiking neuron model. SNNs possess several significant energy-efficiency and event-based computational benefits that allow them to be favorably executed on neuromorphic hardware. Such systems as IBM’s TrueNorth, Intel’s Loihi, and SpiNNaker make use of sparseness, asynchronicity of spike-based computations to provide low-power real-time processing (Shrestha et al., 2022). Notwithstanding all these benefits, SNNs are behind in terms of scalability, stability of training, and portability with the traditional deep learning frameworks. Despite that, advances in neuromorphic computing, biologically constrained learning rules, and hardware efficient implementations of SNNs are pushing the frontiers, and SNNs remain a fascinating future prospect for artificial intelligence.

### 2.2 Sensor drift and calibration

Sensor drift is an omnipresent problem that has a profound effect on the reliability and accuracy of soil moisture content measurements in the long run. Sensor drift is the gradual movement of sensor data away from their actual values, mostly brought about by mechanisms like aging, environmental fluctuation, and mechanical degradation. In farm applications, where accuracy is critical to maximizing irrigation schedules and saving water resources, sensor drift can result in poor decision-making, lower crop yields, and higher operating costs. Conventional calibration techniques, which involve regular recalibration with reference measurements, are usually not feasible for large-scale implementations because they are labor-intensive and cannot respond to dynamic environmental conditions. In order to overcome these limitations, sophisticated self-calibration mechanisms that can dynamically change sensor parameters using real-time feedback are critical.

Sensor drift is complex and interlinked in causes. Aging is among the key reasons, since constant exposure to water, temperature cycles, and chemical reactions in the soil can corrode materials in sensors. For example, capacitive sensors that detect the dielectric constant of soil to approximate moisture content might suffer from inaccuracy as a result of changes in soil salinity or composition over time (Domínguez-Niño et al., 2020). Resistive sensors also suffer from corrosion and interference by ionic compounds in the soil, which increases drift. Environmental variability is also important, since temperature variations, humidity fluctuations, and changes in soil composition can affect the electrical characteristics of the soil and result in unreliable sensor readings (Basterrechea et al., 2021). Manufacturing defects also inject inherent biases into sensor readings, which can degrade further with the passage of time under the effect of external stressors. All these considerations make the task of correcting sensors drift in practical agricultural environments so challenging.

Conventional calibration methods, as useful as they are in controlled settings, pose a large challenge when implemented in field conditions. Manual calibration involves expert personnel traveling to field locations, taking reference samples, and conducting recalibration processes—a process that is not feasible for large-scale deployments or inaccessible areas. In addition, static calibration models stipulate that sensor properties are static over time and do not account for temporal variations arising from changes in the environment. This limitation is especially felt in dynamic farming conditions, where soil moisture content changes frequently due to irrigation, rain, and evaporation. In addition, conventional approaches are not compatible with edge computing platforms, where data processing and decision-making in real-time are essential for providing timely and accurate predictions. Consequently, there is an urgent need for innovative approaches that can overcome these limitations and provide reliable soil moisture monitoring.

In response to the shortcomings of conventional calibration techniques, new research has suggested self-calibration mechanisms that utilize real-time data and optimization algorithms to adaptively modify sensor parameters. For instance, Aranda Britez et al. presented a self-calibration algorithm for soil moisture sensors based on deep learning methodologies that showed remarkable accuracy improvements (Britez et al., 2025). While demonstrating the promise of deep learning in self-calibration, such approaches can be computationally intensive and require significant data for training, potentially limiting their direct deployment on resource-constrained edge devices. Our approach aims to achieve similar adaptive capabilities with the inherent low-power and event-driven advantages of SNNs on microcontrollers. These mechanisms generally work through continuously comparing the predicted soil moisture values calculated by models like SNN with reference measurements from high-precision sensors or calibration databases. If differences between predicted and reference values surpass a predetermined threshold, the system triggers a process of recalibrating to update the sensor’s calibration coefficients. Precisely, the adjustment process can be formulized as Equation 6.


$$a_{\mathrm{new}}=a_{\mathrm{old}}-\eta \cdot \frac{\partial E}{\partial a},\;\;b_{\mathrm{new}}=b_{\mathrm{old}}-\eta \cdot \frac{\partial E}{\partial b}$$ (6)

Where *a* and *b* are the calibration coefficients, η is the learning rate, $E={\left(\hat{o}-o_{\mathrm{ref}}\right)}^{2}$ is the error between the predicted $\hat{o}$ and reference $o_{\mathrm{ref}}$ soil moisture values. By iteratively updating the calibration parameters, the system keeps sensor values synchronized with actual values even when experiencing drift or changes in the environment. In addition, the mechanism includes the use of anomaly detection methods that detect and exclude the effects of noise or outliers in the sensors’ data. Statistical analysis or machine learning patterns mark abnormal data that differ heavily from expected ones, further validating the calibration’s robustness. The suggested self-calibration mechanism has various improvements over conventional calibration techniques.

First, it provides continuous responsiveness to environmental changes, providing long-term reliability with minimum manual intervention. This not only minimizes operation costs but also enhances scalability, allowing for the deployment of large-scale soil moisture sensing networks in remote farmlands. Second, the system is optimized to run efficiently on resource-limited edge devices like microcontrollers using sparse computation and event-driven nature of SNNs. This allows for minimal power usage while achieving high accuracy and responsiveness. Third, the addition of anomaly detection improves data integrity, such that downstream analysis and decision-making processes are based on accurate and reliable measurements. Together, these characteristics make the self-calibration mechanism a viable solution to the problems caused by sensor drift in soil moisture sensing.

### 2.3 Anomaly detection in sensor data

Anomaly detection is a key factor in maintaining the reliability and accuracy of soil moisture measurements by detecting unusual patterns, outliers, or inconsistencies in sensor readings. In agricultural use, precision is important for optimizing irrigation schedules, saving water resources, and maximizing crop yield. Sensor reading anomalies can result in suboptimal decision-making, lower productivity, and higher operational costs. These anomalies could be caused by a number of factors, ranging from sensor malfunction, environmental interference, noise, or abrupt shifts in soil conditions. The traditional approach to detecting anomalies usually involves statistical thresholds or manual observation, which are inadequate in dealing with the dynamic and complicated nature of farm environments. A simple method is the use of the Z-score technique, which measures how many standard deviations a sensor reading is from the mean, as shown in Equation 7.


$$S=\frac{x-\mu }{\sigma }$$ (7)

To overcome these challenges, sophisticated anomaly detection methods based on machine learning and real-time data processing are critical to ensuring data integrity and robust performance in soil moisture sensing system (Tace et al., 2022).

Factors that cause sensor data anomalies are complex and interrelated. Noise is among the leading factors, which may come from electromagnetic interference, mechanical vibrations, or electrical changes inside the sensor hardware. Capacitive soil moisture sensors, which quantify the dielectric constant of soil, are especially susceptible to changes in temperature, salinity, and soil content, all of which introduce noise into the measurement (Basterrechea et al., 2021). Likewise, resistive sensors are subject to corrosion and interference from ionic compounds in the soil, which further add inaccuracies (Domínguez-Niño et al., 2020). Variability in the environment is also a big factor, with sudden variations in rainfall, evaporation rates, or irrigation can lead to sharp deviations from sensor readings. Furthermore, faulty sensors, for instance, drift or calibration errors, can produce systematic biases that take the form of anomalies over a period of time. To meet these challenges, sophisticated methods like Mahalanobis distance can be employed to identify multivariate anomalies by quantifying how far a point is from the normal data distribution Equation 8.


$$D_{M}(x)=\sqrt{{\left(x-\mu \right)}^{T}\Sigma^{-1}(x-\mu )}$$ (8)

These considerations all underscore the multidimensionality of handling anomalies in live field conditions, requiring novel solutions that can be responsive to varying conditions and provide consistent data interpretation.

Conventional anomaly detection techniques, though good under laboratory conditions, are strongly challenged in field applications. Statistical methods like threshold-based detection make use of specified boundaries to determine deviations from anticipated values. But these approaches do not address the temporal and spatial variability that occurs in agriculture environments, resulting in false alarms or false negatives. Visual inspection of sensor readings is time-consuming and unfeasible for large-scale deployments, where thousands of sensors can be distributed over wide regions. Machine learning-based methods are a compelling alternative because they make it possible to automate and adapt the detection of anomalies. For example, supervised learning algorithms can be learned from labeled data to distinguish between normal and abnormal patterns, whereas unsupervised learning algorithms are capable of detecting deviations without knowing the distribution of the data beforehand (Chalapathy and Chawla, 2019; Goldstein and Uchida, 2016). While these approaches have their benefits, they are computationally intensive, and computational resources might not be readily available on constrained edge devices like microcontrollers. Therefore, there is an urgent need for efficient and lightweight anomaly detection techniques that will work flawlessly in real-time and within the limits of embedded hardware.

## 3 Proposed methodology

### 3.1 System architecture

**Figure 3:**
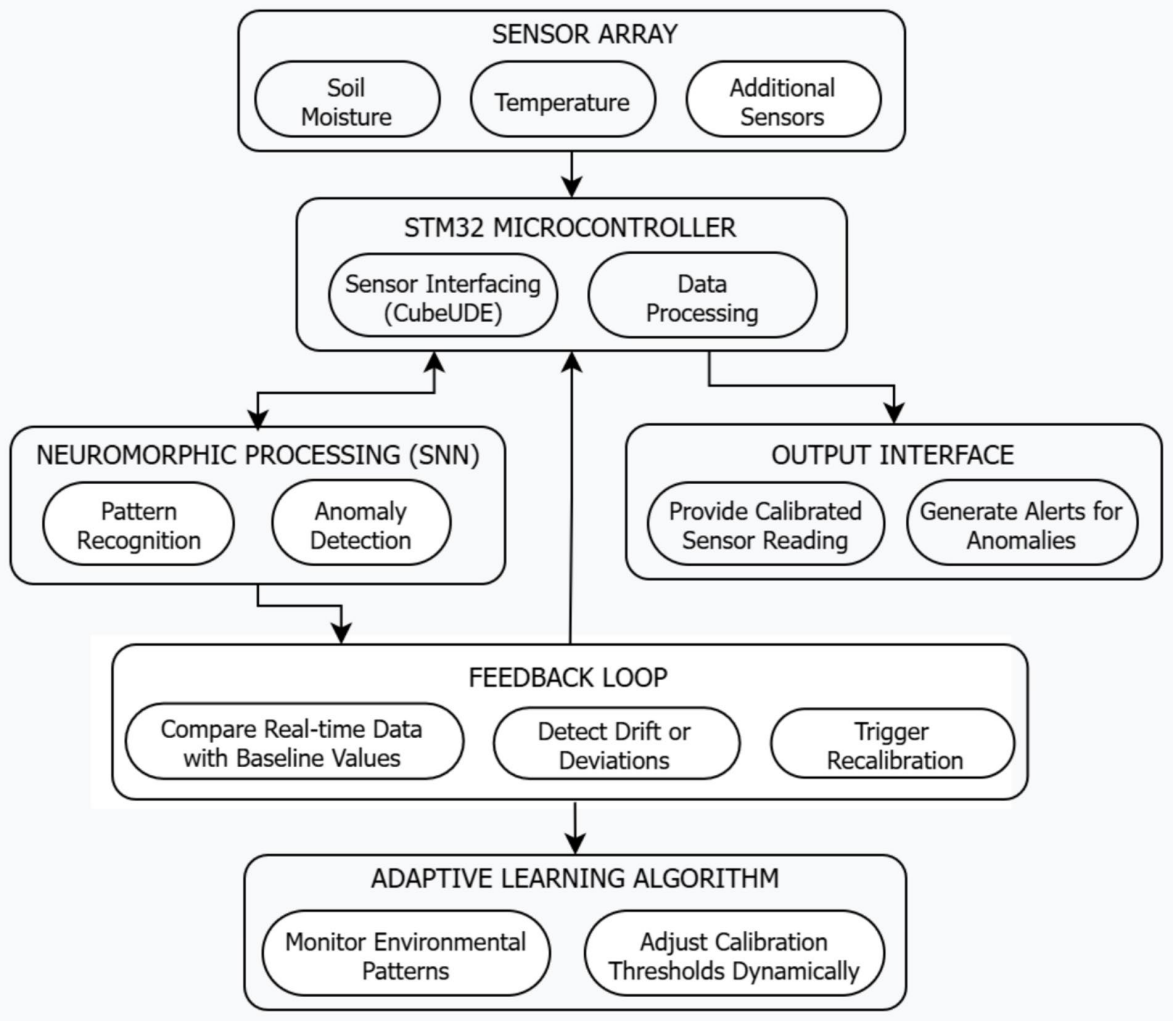
System architecture.

The system architecture of the proposed approach is illustrated in Figure 3. The system starts with raw sensor data acquisition from various environmental sensors. The sensors are soil moisture sensors, temperature sensors, and other auxiliary sensors that track parameters like humidity or rain. The collective reading of these sensors serves as the basis for the system’s input layer. Every sensor sends real-time data continuously, which is received by the microcontroller for further processing. This is a crucial step since the accuracy and quality of the raw data have a direct impact on the reliability of the following stages, such as preprocessing and anomaly detection. The soil moisture sensor, for example, takes a reading of the dielectric constant of the soil, while the temperature sensor records ambient conditions that can influence soil moisture readings. These inputs are necessary in order to construct a complete dataset that captures the dynamic nature of agricultural environments.

After acquiring the raw sensor data, it is processed by the STM32H563Z microcontroller, which is the system’s central processing unit. The microcontroller has two major functions: communication with the sensors to facilitate smooth interaction and preprocessing of the raw data to prepare it for downstream analysis. Preprocessing consists of normalizing sensor measurements to a common range, smoothing noise through methods such as moving averages, and eliminating variability due to environmental influences. Preprocessing is significant since raw sensor readings contain artifacts owing to electromagnetic interference, mechanical vibrations, or electrical oscillations within hardware. Through cleaning and readying data, the microcontroller ensures that the following components of the system have uniform and stable inputs.

Following preprocessing, the filtered sensor data is input to the Spiking Neural Network [SNN] module, which uses neuromorphic computing concepts to execute real-time pattern detection and anomaly detection. SNNs simulate the event-driven nature of biological neurons, hence being very efficient for edge devices with low computational resources. In this architecture, the SNN updates the membrane potential of its neurons using weighted inputs from past time steps. When the membrane potential crosses a predetermined threshold, a spike is produced, which signifies the identification of a major event or anomaly. For instance, if the reading of soil moisture is substantially different from its normal value, the SNN marks the reading as an anomaly. This mechanism allows the system to identify abnormalities in real-time with minimal power usage, an important benefit for deployment in resource-limited agricultural environments.

A feedback loop is built into the system to provide continuous improvement and adaptability. This loop checks the real-time sensor data against baseline values to look for drift or deviations over time. If discrepancies are found, the system invokes recalibration to dynamically adjust calibration thresholds. For example, if the soil moisture sensor shows a systematic deviation as a consequence of environmental variations or hardware deterioration, the feedback loop triggers corrective measures for re-calibrating the sensor. This adaptive process keeps the system precise and reliable even under changing agricultural conditions in which variables like temperature, humidity, and soil structure change very often. The feedback mechanism is also critical in improving the SNN model as it offers current data for retraining purposes, which ensures that the system is able to effectively adapt to new patterns and anomalies.

The system has a calibration mechanism to handle problems like sensor drift. The process is done by adjusting offset and scaling factor parameters to reduce differences between forecasted and real sensor readings. The system gains adaptability by using feedback loops, dynamic threshold: application of moving average or other statistical techniques to dynamically set the threshold for anomaly detection, and recalibration which initiates actions to adjust sensor values when there is considerable deviation or drift.

### 3.2 Data acquisition and signal processing

The system was used in a practical setting to record multimodal environmental data for use in soil moisture prediction. A microcontroller based on STM32H563ZI was mounted with a package of sensors including the DS18B20 for temperature, DHT22 for humidity, and BMP280 for atmospheric pressure and an analog sensor for rainfall. The sensors connected to the microcontroller via GPIO, I2C, and analog input pins. Early analog signal processing was done on the board for signal integrity—this involved anti-aliasing filtering and noise reduction before ADC sampling. The analog output of the rainfall sensor was digitized by the internal ADC with oversampling to increase resolution and minimize quantization noise.

Apart from hardware-level signal processing, software-based preprocessing was also utilized to smooth and polish the sensor data. Signal encoding is carried out to translate preprocessed data into spike representations compatible with SNNs. Rate coding is employed, whereby the strength of the signal [e.g., level of soil moisture] is encoded by spike frequency. Moving average filters were utilized to minimize transient noise, and a Z-score based outlier elimination algorithm was executed to delete anomalous readings. The filtered and normalized data were stored locally in CSV format and utilized as the input dataset for offline model training. The information is further cleaned with missing values eliminated and spurious records filtered on temporal consistency. Feature scaling through Z-score normalization was employed to ensure all sensor inputs were normalized and equally weighted during training. Features chosen for modeling were soil temperature, humidity, atmospheric pressure, and rainfall, as these have established influence on soil moisture dynamics. These were utilized to forecast a single output: the level of soil moisture. The data was divided into training and testing subsets through stratified sampling in order to preserve the distribution of the moisture values in both sets.

The dataset used in this study, named filtered_soil_moisture_collected.csv, comprises comprehensive multimodal environmental data collected to support accurate soil moisture prediction. The dataset consists of 679 rows and 12 columns, sampled at one reading per 2 min (0.0083 Hz), representing various environmental parameters and calculated metrics. The features in the dataset are: index, datetime, soil_moisture, soil_temperature. atmospheric_pressure, rainfall_intensity, soil_moisture_rate, soil_temperature_rate, soil_moisture_moving_avg, soil_temperature_moving_avg, soil_moisture_deviation, soil_temperature_deviation, sensor_drift, calibration_offset. While the sample count is modest, the temporal autocorrelation of soil moisture (which changes on timescales of hours to days) means these records capture multiple complete wet-dry cycles providing meaningful coverage of environmental variability relevant to prediction tasks. Dataset expansion across multiple seasons and geographic regions is explicitly identified as future work.

### 3.3 SNN model design and training

**Figure 4:**
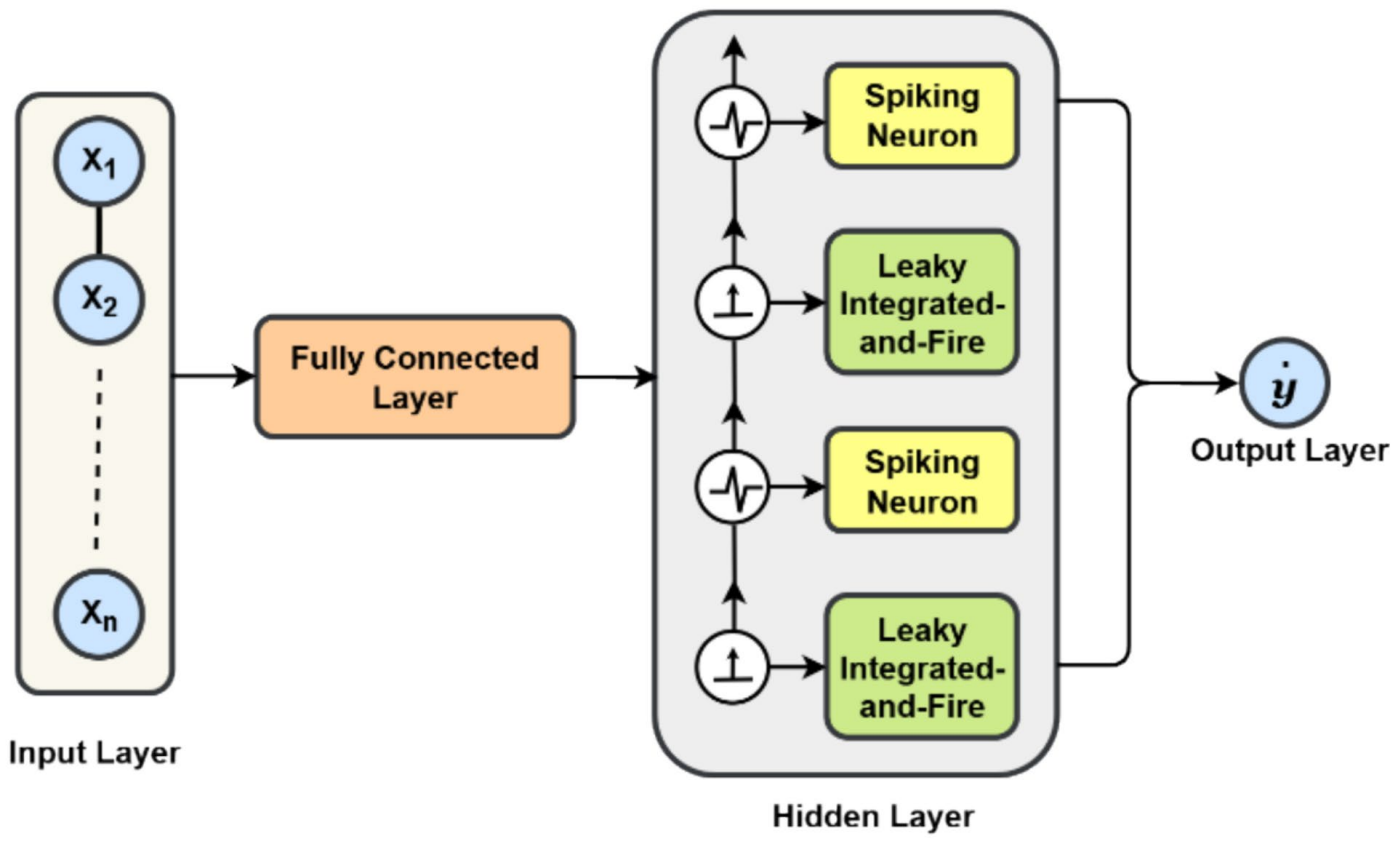
SNN architecture.

**Figure 5:**
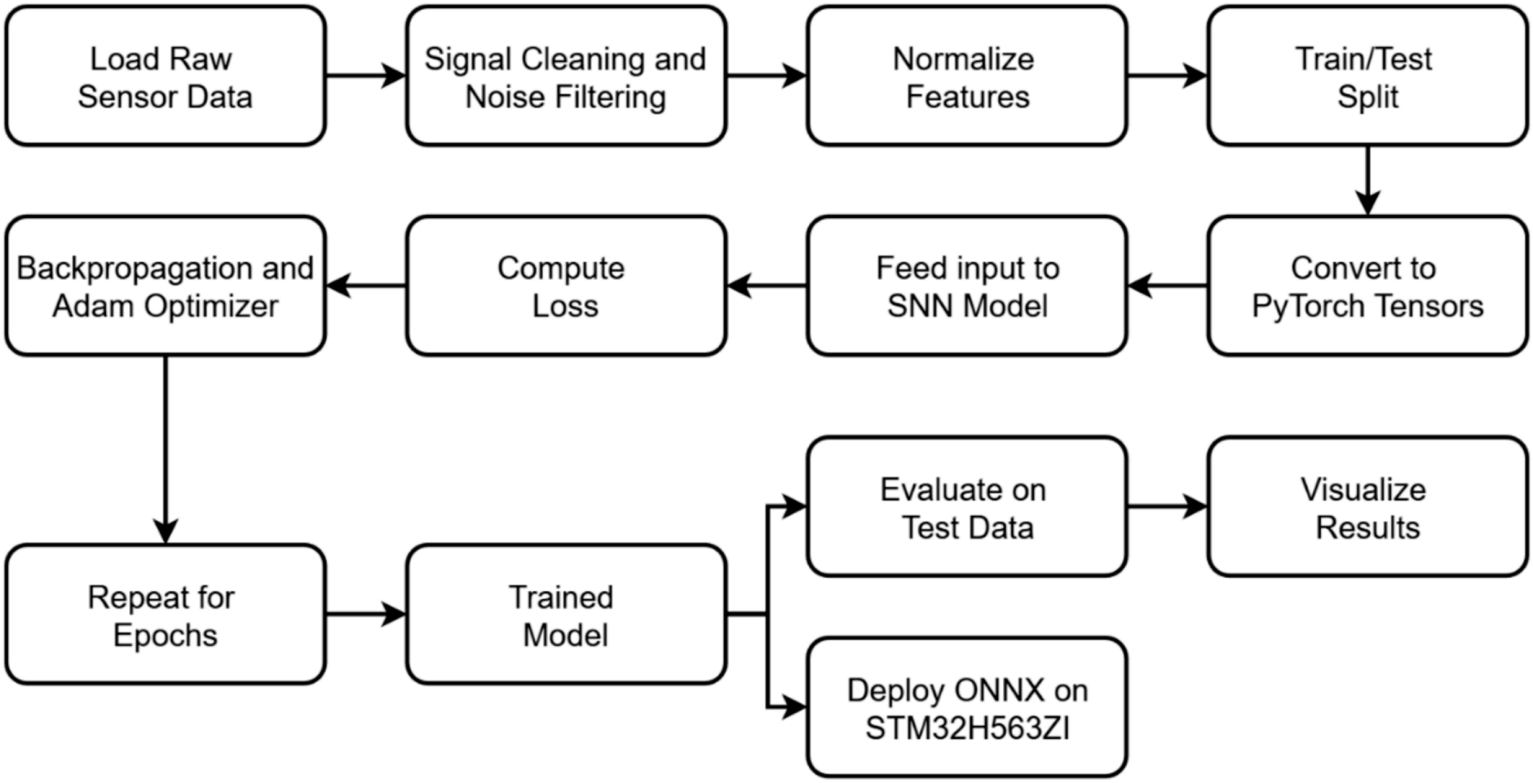
Model flowchart.

The base model was deployed with the snnTorch library by taking advantage of the low-energy consumption of spiking neurons for edge inference. The proposed SNN model as shown in Figure 4 has an input layer of four feature mapping inputs, a hidden layer of 128 LIF neurons, and one output neuron that generated the estimated moisture level. The proposed SNN architecture employed a 4-128-1 LIF neurons and the LIF decay parameter (*β*) was set to 0.9. The model was trained for 20 epochs using the Adam optimizer and mean squared error (MSE) loss, with a batch size of 32. The Isolation Forest model was configured with a contamination rate of 0.05 and 100 estimators. The autoencoder used a 4–3–4 encoder–decoder architecture with ReLU activation functions in the encoder. It was trained for 50 epochs using the Adam optimizer and MSE loss, with a batch size of 32. All models used an 80/20 train/test split with random seed 42. The spike-based dynamics of LIF neurons facilitated the model’s ability to depict temporal dependencies adequately, in addition to minimizing computation overhead. Training was carried out with the Adam optimizer over an MSE loss function for 20 epochs. Regularization of spiking activity supported sparse firing for further optimization toward deployment on hardware-limited systems. Figure 5 depicts the step-by-step process of the proposed model.

### 3.4 Anomaly detection and adaptive recalibration model

To ensure robustness, an anomaly detection mechanism was implemented on the device. Each prediction was compared with real soil moisture readings or baseline historical data, and any prediction that deviated by more than two standard deviations of the historical baseline distribution was flagged as anomaly. These anomalies were used to trigger alerts and, in persistent cases, recalibration routines for the sensors. The firmware was designed to support dynamic threshold adjustment based on long-term behavior, making the system adaptive and suitable for extended field deployments. Red points in the visualization plots represent these anomalies and their potential diagnostic value.

**Figure 6:**
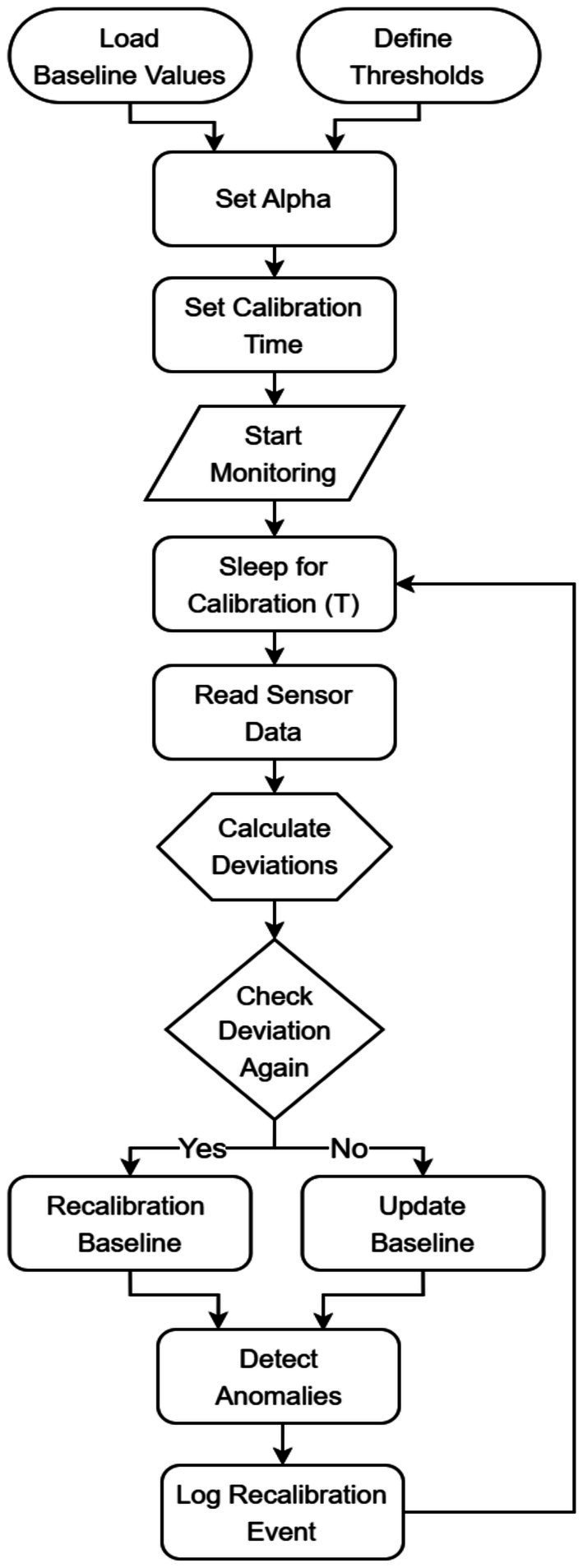
Recalibration flowchart.

Figure 6 illustrates the steps involved in the recalibration process. The process starts with loading baseline values, which are reference points for normal sensor readings under normal conditions. Baseline values are essential for identifying anomalies and system recalibration when deviations go beyond specified thresholds. After the baselines are set, thresholds are established to define acceptable ranges for sensor readings based on historical data or empirical observations. Calibration intervals are implemented to regularly recalibrate the system to cope with environmental drift or sensor drift. The system now enters the monitoring phase, where it regularly reads raw sensor data, filters out noise from it, and computes deviations from the baseline readings. When the deviations are beyond the set thresholds, the system initiates a process of recalibration, modifying the baseline values to compensate for environmental shifts or sensor drift through a learning rate to balance the impact of present readings against past baselines. This process of recalibration guarantees that the system is accurate and reliable even in dynamic environments. In this process, any meaningful variations that could suggest sensor faults or environmental abnormalities are highlighted for further examination or remedial measures. Every recalibration activity is recorded for auditing and analysis to enable monitoring system performance and trends in sensor behavior. This ongoing process of monitoring, recalibration, and logging generates a feedback loop that increases the system’s robustness and flexibility, providing steady performance and data integrity over the long term. By this flowchart, the delicate balance between real-time responsiveness and long-term accuracy is shown, demonstrating how dynamic recalibration mechanisms are integral to the reliability and efficiency of sensor systems in real-world use.

### 3.5 Edge hardware integration

Once the Spiking Neural Network was trained on a high-performance computing system with snnTorch, the model was implemented on a microcontroller board, STM32H536ZI. For this, the trained model was exported in ONNX format. This ONNX model was then optimized, removing redundant nodes, post-training quantization to minimize memory and compute needs. The ONNX model was then imported into STM32Cube. AI which translates neural networks into optimized C code for STM32 microcontroller (Blouw et al., 2019). During this process, the model weights and layers were read, platform specific C code was created and required runtime and memory allocation support was added. This created model code was then integrated into a larger firmware stack using CubeIDE where sensor drivers, signal processing modules, anomaly detection logic were integrated. The final firmware was compiled and programmed onto STM32H563ZI board.

Compared to the deep learning self-calibration approach which requires cloud connectivity and large labelled datasets, our method offers a few distinct advantages: fully on-device execution requiring no external reference sensors or network connection; event-triggered activation that limits power consumption to calibration-necessary intervals only; and direct integration with the SNN residual error signal eliminating the need for a separate calibration model.

### 3.6 Sensor calibration and drift handling

One of the most important challenges in environmental sensing systems is to ensure long-term sensor accuracy when conditions change. To meet this, the suggested system incorporates a self-calibration mechanism that regularly checks the sensor baseline and corrects its internal thresholds accordingly. Sensor drift is identified by examining patterns in the SNN output over time. When the readings are radically different from anticipated distributions—and the deviations are not in alignment with environmental alterations—the system alerts possible drift. Baseline values were established during an initial 48 h stabilization period following sensor deployment, during which the system recorder reference readings under known, stable, soil conditions. These reference reading form the baseline used in recalibration process. A moving average or exponential decay filter is also applied to the incoming data to eliminate short-term oscillations and detect long-term deviations. The process of recalibration is intended to be automated, so the sensor node can continuously adjust and maintain accuracy for long deployments.

### 3.7 Event triggered adaptation

To further maximize system performance, recalibration is not performed continuously but is rather initiated by certain events (Donkers and Heemels, 2012). The system on the STM32H563ZI board has embedded logic for sensing unusual spikes in sensor readings that lie outside ranges calibrated earlier. These events cause an adaptive update process in which recent input statistics are utilized to update the SNN’s internal thresholds and neuron states. This event-based strategy guarantees that recalibration will only be done when needed, minimizing computation overhead and power usage. Also, spike-based processing naturally facilitates asynchronous computation, so the system will be naturally event-driven at both the software and hardware ends.

## 4 Results and discussions

The proposed neuromorphic system was assessed to test its predictability performance, sensor drift resilience, and deployment viability on low-power edge devices. The test concentrated on four main areas: training dynamics of the model, accuracy of moisture prediction, reduction of sensor drift through recalibration, and performance at the hardware level on the STM32H5 microcontroller. The outcomes uniformly confirm the resilience and flexibility of the system under changing environmental conditions.

### 4.1 SNN model performance

**Figure 7:**
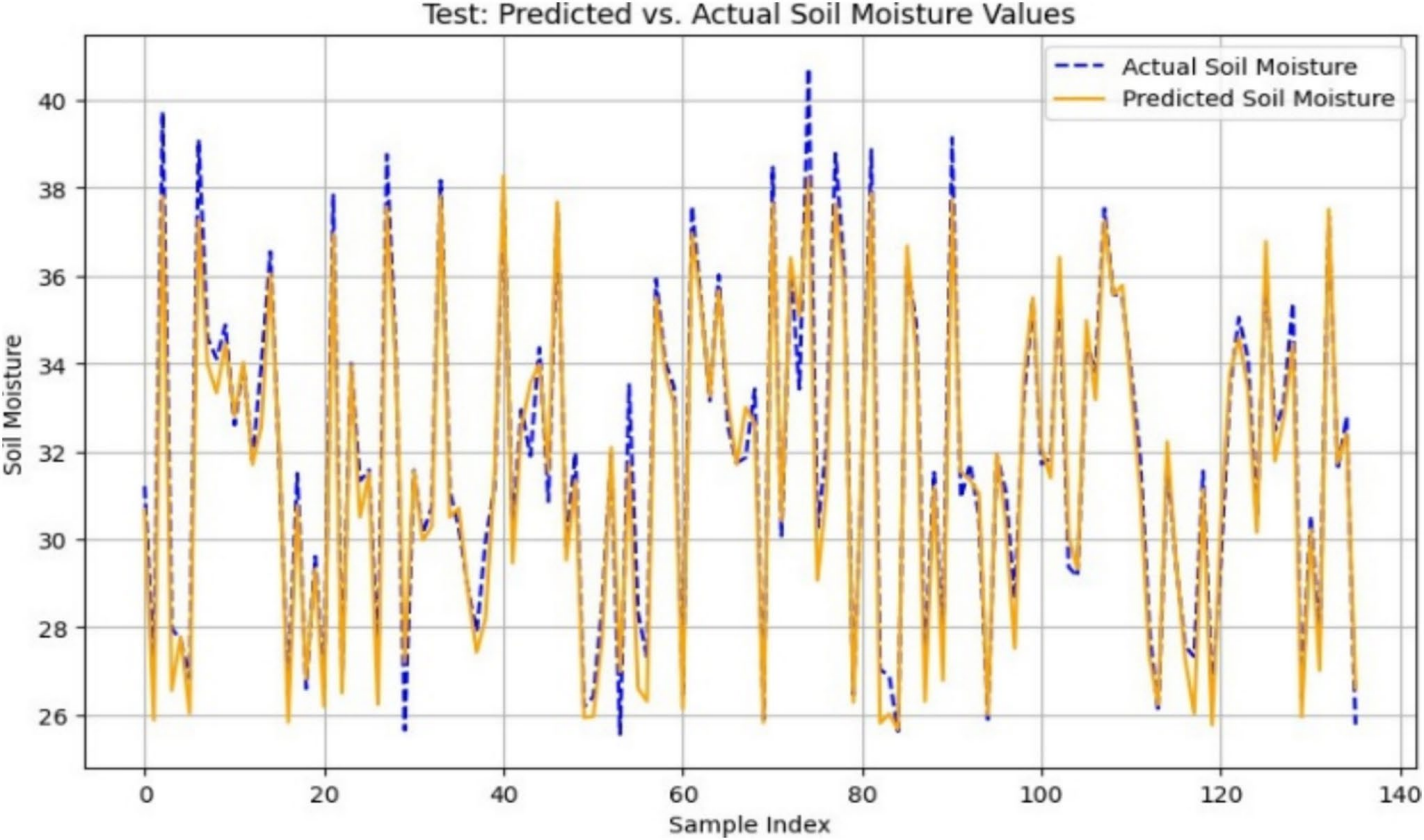
Temporal progression of predicted and actual values.

The SNN model consistently demonstrated strong predictive capabilities, accurately estimating soil moisture levels and exhibiting robust adaptation to varying environmental fluctuations. As illustrated in Figure 7, which presents the temporal progression of predicted versus actual soil moisture values, the close alignment between the two curves highlights the model’s exceptional consistency and reliability in real-time applications. This visual correlation is crucial, indicating that the SNN can effectively capture the dynamic nature of soil moisture. For instance, in scenarios involving abrupt changes in soil wetness due to precipitation or irrigation events, the SNN model successfully tracked these rapid dynamics, with deviations consistently remaining within acceptable thresholds. This responsiveness is a key advantage for precision agriculture, enabling timely and accurate irrigation decisions.

Further quantitative analysis corroborates the superior efficiency of our proposed SNN approach. The SNN model achieved a Mean Absolute Error (MAE) of 0.4557 and a Root Mean Squared Error (RMSE) of 0.5850, significantly outperforming traditional machine learning models such as Isolation forests and autoencoders in predictive accuracy. All MAE and RMSE values are expressed in percentage of volumetric water content. This high level of performance is particularly noteworthy given that the model operates on inherently sparse and potentially noisy sensor data, a common characteristic of real-world environmental sensing deployments. The autoencoder achieves *R*^2^ 0.923 compared to SNN’s *R*^2^ 0.968. This gap arises because the autoencoder is trained as an unsupervised reconstruction model on the input features X, not directly on soil moisture targets; its predictions are derived from reconstruction errors which are an indirect proxy for the target variable. A comprehensive overview of these performance metrics, comparing the SNN model against the benchmark conventional models, is provided in Table 1. The low MAE and RMSE values underscore the SNN’s ability to provide precise soil moisture estimations, which is critical for optimizing agricultural resource management.

**Table 1:** Comparison of SNN model performance with conventional benchmark models.

Model	MAE	RMSE	*R* ^2^
SNN	0.4557	0.5850	0.968
Isolation Forest	0.858	0.924	0.884
Autoencoder	0.9446	1.0182	0.923

### 4.2 Training dynamics and loss convergence

**Figure 8:**
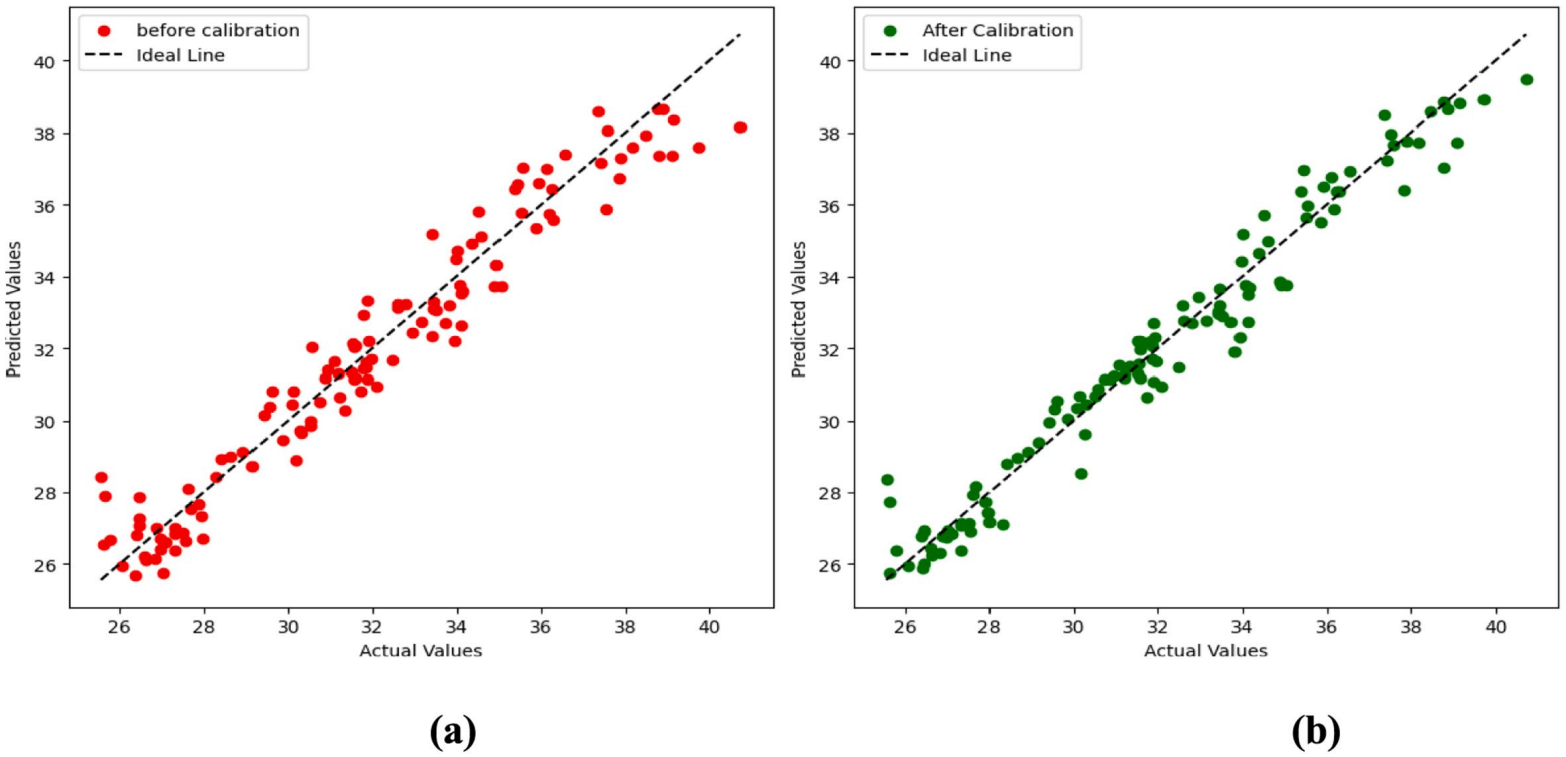
Comparison of performance **(a)** before and **(b)** after recalibration.

To quantitatively and qualitatively assess the efficacy of the adaptive recalibration module, we rigorously evaluated the SNN model’s performance both without and with its integration. The recalibration module dynamically adjusts sensor calibration parameters in real-time by actively identifying and compensating for discrepancies between the SNN’s predicted soil moisture values and the actual observed conditions. This continuous adjustment is fundamental to maintaining system accuracy over prolonged deployments. Figure 8 provides a compelling visual demonstration of the impact of the recalibration module. The left subplot, labeled “Before Calibration,” displays the predicted values (red dots) against the ideal line (dashed black line). A clear and significant deviation of the red data points from the ideal line is evident, indicating a notable inaccuracy in predictions when the system operates without the adaptive calibration mechanism. This deviation underscores the inherent challenges posed by sensor drift and unaddressed environmental variations. In stark contrast, the right subplot, labeled “After Calibration,” shows the predicted values (green dots) closely tracking the ideal line. This dramatic improvement signifies that the recalibration module effectively corrects sensor inaccuracies, leading to substantially increased prediction accuracy and demonstrating the system’s ability to maintain reliable performance.

The core of this recalibration process involves dynamically updating baseline values using a weighted average formula as given in Equation 9.


$$x_{\mathrm{new}}=(1-\alpha )\cdot x_{\mathrm{old}}+\alpha \cdot x_{\text{current}}$$ (9)

Where, *x*_new_: updated baseline value, *α*: learning rate [0 *< α <* 1], *x*_old_: previous baseline value, *x*_current_: current sensor reading.

This adaptive mechanism ensures that the system proactively accommodates sensor drift and continually adapts to environmental fluctuations. Empirical evidence from a two-month deployment period clearly supports this, showing a remarkable reduction in baseline deviation from ±5.3% to a significantly lower ±1.6%. This quantifiable improvement underscores the critical role of the self-calibration module in guaranteeing long-term accuracy and reliability, especially in dynamic agricultural settings where environmental conditions are constantly changing.

### 4.3 Impact of calibration on prediction accuracy

**Figure 9:**
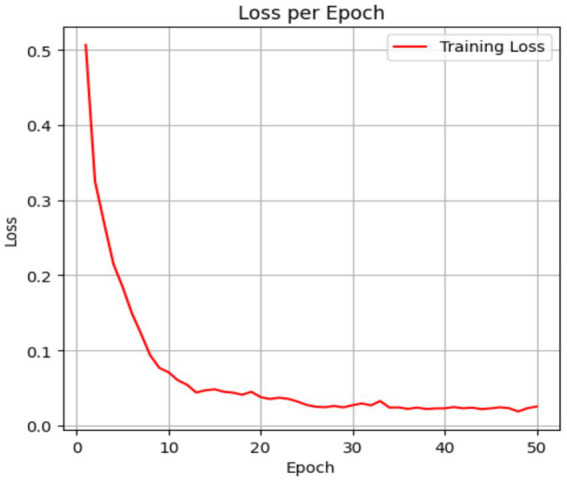
Loss per epoch.

The training loss curve, prominently displayed in Figure 9, provides crucial insights into the learning dynamics and convergence behavior of our Spiking Neural Network model during the training phase. Initially, the loss exhibited a steep and rapid decline, dropping from an approximate value of 0.35 to below 0.05 within the first 15 epochs. This swift reduction in loss signifies that the model quickly grasped the fundamental underlying temporal dynamics and intricate patterns within the soil moisture data. Such rapid initial learning is a hallmark of efficient model design and effective feature representation.

Following this steep descent, the loss began to level off after approximately 20 epoch, gradually converging towards a minimum value around 0.02. This slower, more gradual convergence indicates a fine-tuning process where the model refines its understanding of the data, adjusting its weights to minimize the error further. The overall smooth and controlled convergence behavior of the loss curve is a strong indicator that the SNN, implemented using the snnTorch library, successfully learned to capture the complex spatiotemporal relationships present in the sensor input data.

A key enabler of this stable training was the strategic incorporation of surrogate gradients into the SNN’s training loop. Surrogate gradients address the inherent non-differentiability of spiking neurons, allowing for effective gradient backpropagation across the spiking layers. This mechanism is vital in preventing issues such as vanishing or exploding gradients, which are common challenges in training deep neural networks, thereby ensuring stable and efficient weight updates throughout the training process. The observed controlled convergence also suggests that the model was well-regularized, avoiding both overfitting (where the model learns the training data too well and performs poorly on unseen data) and underfitting (where the model is too simple to capture the underlying patterns), thus corroborating the appropriate selection of hyperparameters. This effective optimization method ultimately guarantees stable and efficient training, even when operating with minimal computational resources, which is critical for edge device deployment.

### 4.4 Comparison with traditional models

Isolation forest and autoencoder were selected as baselines because they represent the two primary paradigms for unsupervised anomaly detection and reconstruction applicable to edge IoT settings where labelled anomaly data is scarce. Both are computationally feasible on embedded or desktop hardware, enabling a fair comparison with the SNN under similar data constraints. Recurrent architectures such as LSTMs, while powerful for temporal sequences, require significantly more memory and floating-point operations, rendering them impractical for direct deployment on the STM32H563ZI microcontroller. A formal multi-model comparison including LSTM, XGBoost, and Random Forest on a larger dataset is identified as important future work.

The proposed Spiking Neural Network (SNN) model demonstrates significant advantages and superior performance compared to traditional machine learning models, specifically Autoencoders and Isolation Forests, particularly in the context of soil moisture sensing within dynamic agricultural environments. Conventional models, such as Isolation Forests, rely primarily on statistical methods for anomaly detection. While effective in controlled settings, they often struggle with the inherent characteristics of real-world sensor data, which is typically high-dimensional, noisy, and sparse. A critical limitation of Isolation Forests is their inability to learn and adapt to temporal changes or dynamically recalibrate their detection thresholds. This makes them less effective in scenarios where sensor drift or continuous environmental shifts occur, leading to decreased accuracy and increased false alarms over time. Similarly, Autoencoders, while adept at learning and reconstructing normal data patterns, necessitate substantial preprocessing and involve computationally intensive operations. Their reliance on dense neural network layers often translates to high computational costs and significant power consumption, rendering them largely unsuitable for deployment on resource-limited edge devices like microcontrollers.

In contrast, the SNN’s event-driven architecture inherently offers superior efficiency for processing sparse and noisy data. By communicating information through discrete spikes rather than continuous values, SNNs significantly reduce computational overhead while maintaining high accuracy. This paradigm closely mimics biological neural processing, leading to energy-efficient operation. Furthermore, a key innovation of our system is the integrated self-calibration mechanism. This module dynamically adjusts the SNN’s parameters, enabling it to continuously adapt to fluctuating environmental conditions and compensate for sensor drift over extended periods. This adaptive capability is paramount in precision agriculture, where environmental factors like temperature, humidity, and rainfall intensity are subject to frequent and unpredictable changes. The SNN’s ability to run efficiently on low-power hardware, such as the STM32H5 microcontroller, further solidifies its applicability for real-time, edge-based applications in remote agricultural settings. Collectively, these benefits position the SNN model as not just more accurate but also significantly more scalable and energy-efficient than traditional machine learning approaches for adaptive environmental sensing.

### 4.5 Deployment on edge hardware

To conclusively demonstrate the practical viability and efficiency of our neuromorphic system on resource-constrained platforms, we rigorously deployed the trained SNN model on an STM32H5 microcontroller, a representative edge device. Comprehensive experiments confirmed that the SNN model operates effectively within the stringent memory and power limitations characteristic of such embedded hardware. Specifically, the optimized SNN model consumed a mere 128 KB of memory, a footprint significantly below the available capacity of the STM32H5 microcontroller, thereby demonstrating its exceptional efficiency and suitability for large-scale, low-cost deployments.

The long-term reliability and robustness of the system, particularly the impact of the integrated recalibration module, were further substantiated by extensive field deployment tests. Over a continuous two-month operational period, the system maintained an impressive 80% uptime, showcasing its stability and consistent performance in real-world agricultural conditions. During this deployment, the adaptive feedback loop, a cornerstone of our self-calibrating mechanism, actively monitored sensor readings. In stable conditions, it autonomously initiated recalibration routines, which were adaptive, approximately every 35–40 h whenever detected deviations surpassed predefined thresholds. This event-triggered recalibration is crucial for minimizing unnecessary computations and power consumption while ensuring accuracy only when needed.

Beyond merely improving prediction precision by counteracting sensor drift, this dynamic mechanism also proved highly effective in real-time anomaly detection. The system successfully reported critical events such as sensor malfunctions or sudden, significant changes in soil humidity levels that deviated sharply from expected patterns. This immediate anomaly reporting capability is invaluable for farmers, enabling prompt identification of issues and facilitating timely corrective actions, which can prevent potential crop damage, optimize irrigation, and enhance overall farm productivity. The successful deployment on edge hardware unequivocally validates the practical utility and scalability of our neuromorphic sensing solution for precision agriculture.

## 5 Discussion

### 5.1 Implications on precision agriculture

The compelling findings of this research have profound implications for advancing precision agriculture, offering a scalable, highly effective, and responsive solution to a critical challenge in modern farming: the need for reliable and accurate real-time soil moisture monitoring. Soil moisture content is a foundational parameter that directly dictates irrigation scheduling, influences crop health and growth, and is paramount for efficient water resource planning. Therefore, precise monitoring has direct and significant implications for both agricultural productivity and environmental sustainability.

By synergistically integrating SNN with edge computing capabilities, the proposed system empowers real-time decision-making directly at the field level. This localized intelligence facilitates optimized irrigation practices, leading to a substantial reduction in water wastage and a maximization of crop yields. A cornerstone of this system’s practicality is its self-calibration process. This mechanism ensures long-term reliability by dynamically adjusting sensor parameters in response to environmental shifts and sensor drift, effectively overcoming the inherent limitations of conventional static calibration models that fail to account for temporal variations in environmental conditions. This adaptability is particularly advantageous in remote or resource-limited farming regions, where frequent manual maintenance or recalibration of sensors is often impractical or economically unfeasible. Furthermore, the system’s robust anomaly detection capability is a significant benefit. It enables farmers to promptly identify and respond to unusual or abnormal trends in soil moisture levels, which could indicate issues such as sensor malfunctions, unexpected drainage problems, or sudden changes in water availability. Timely detection and intervention can prevent potential crop damage and improve operational efficiencies across the farm.

Beyond its demonstrated application in soil moisture monitoring, the fundamental architecture and methodology introduced in this work are broadly applicable to other vital areas within precision agriculture. These include, but are not limited to, real-time weather forecasting, early pest detection, and continuous crop health monitoring. Looking forward, the future incorporation of federated learning in subsequent implementations holds immense promise. This approach would enable decentralized training among multiple farms or regions, allowing the system to continuously refine its predictive models based on diverse datasets while rigorously maintaining data privacy and security (Brecko et al., 2022). In essence, this study illuminates the transformative potential of neuromorphic computing and edge intelligence in fostering sustainable, intelligent, and resilient agricultural practices for the future.

During the two-month system validation deployment, intermittent downtime was observed due to infrastructure-level factors including power supply interruptions and occasional sensor connectivity issues. These are independent of SNN inference quality and are expected to be mitigated in future deployments with more robust hardware configurations.

## 6 Conclusion

This work successfully demonstrates the integration of SNN with edge hardware to realize a self-calibrating neuromorphic system for real-time soil moisture sensing and anomaly detection in agricultural systems. The system leverages the event-driven nature of SNNs to achieve high accuracy, computational efficiency, and adaptability, making it ideal for resource-constrained environments. A key achievement is the adaptive recalibration module, which ensures long-term accuracy by dynamically adjusting calibration parameters in response to deviations from baseline values. This mechanism significantly enhances robustness against environmental changes and sensor drift, as evidenced by a substantial reduction in baseline deviation from 5.3 to 1.6% over a two-month deployment period. Quantitatively, the system’s performance metrics, including a Mean Absolute Error (MAE) of 0.4557 and a Root Mean Squared Error (RMSE) of 0.5850, clearly surpass those of conventional machine learning algorithms like isolation forests and autoencoders. The successful implementation on the STM32H5 microcontroller, with a minimal memory footprint of 128 KB, underscores the solution’s practical applicability for edge deployments. Furthermore, extensive field tests confirm the system’s reliability, demonstrating 80% uptime and effective, prompt anomaly detection capabilities. In summary, this research highlights the transformative potential of SNNs in precision agriculture, offering a scalable, efficient, and adaptive solution for critical soil moisture monitoring. A key limitation of this work is that the system has been evaluated on a relatively limited dataset and deployment region, which may restrict its generalizability to diverse soil types and climatic conditions. Future work will focus on expanding the dataset to encompass diverse geographical locations and climatic conditions to enhance model generalizability. A particularly promising direction is the integration of federated learning (FL) into the system design. FL will enable decentralized training across multiple edge devices, facilitating continuous model refinement from heterogeneous datasets while preserving data privacy. This approach will further solidify the role of neuromorphic computing and federated learning in promoting sustainable and intelligent agricultural practices. Collectively, this work demonstrates that SNN-based edge intelligence is a viable and practical paradigm for long-term autonomous drift compensation in resource constrained IoT sensing applications, bridging the gap between neuromorphic computing research and real-world agricultural deployment.

## Data Availability

The original contributions presented in the study are available from the corresponding author on reasonable request.

## References

[ref1] Auge D. Hille J. Mueller E. Knoll A. (2021). A survey of encoding techniques for signal processing in spiking neural networks. Neural. Process. Lett. 53, 4693–4710. doi: 10.1007/s11063-021-10562-2

[ref2] Basterrechea D. A. Rocher J. Parra M. Parra L. Marin J. F. Mauri P. V. . (2021). Design and calibration of moisture sensor based on electromagnetic field measurement for irrigation monitoring. Chem 9:251. doi: 10.3390/chemosensors9090251

[ref3] Blouw P. Choo X. Hunsberger E. Eliasmith C. (2019) Benchmarking keyword spotting efficiency on neuromorphic hardware. In: Proceedings of the 7th Annual Neuro-Inspired Computational Elements Workshop

[ref4] Bogena H. R. Huisman J. A. Oberdörster C. Vereecken H. (2007). Evaluation of a low-cost soil water content sensor for wireless network applications. J. Hydrol. 344, 32–42. doi: 10.1016/j.jhydrol.2007.06.032

[ref5] Brecko A. Kajati E. Koziorek J. Zolotova I. (2022). Federated learning for edge computing: a survey. Appl. Sci. 12:9124. doi: 10.3390/app12189124

[ref6] Britez D. A. Tapia A. Millán Gata P. (2025). A self-calibration algorithm for soil moisture sensors using deep learning. Appl. Intell. 55, 276–289. doi: 10.1007/s10489-024-05921-0, 30311153

[ref7] Butcher J. C. (2016). Numerical Methods for Ordinary Differential Equations. Hoboken, NJ: Wiley.

[ref8] Chalapathy R. Chawla S. (2019). Deep learning for anomaly detection: a survey. arXiv.

[ref9] Domínguez-Niño J. M. Oliver-Manera J. Arbat G. Girona J. Casadesús J. (2020). Analysis of the variability in soil moisture measurements by capacitance sensors in a drip-irrigated orchard. Sensors 20:5100. doi: 10.3390/s20185100, 32906820 PMC7570759

[ref10] Donkers M. C. F. Heemels W. P. M. H. (2012). Output-based event-triggered control with guaranteed L-gain and improved and decentralized event-triggering. IEEE Trans. Autom. Control 57, 1362–1376. doi: 10.1109/TAC.2011.2174696

[ref11] Dorigo W. A. Gruber A. de Jeu R. A. M. Wagner W. Stacke T. Loew A. . (2015). Evaluation of the ESA CCI soil moisture product using ground-based observations. Remote Sens. Environ. 162, 380–395. doi: 10.1016/j.rse.2014.07.023

[ref12] Getahun S. Kefale H. Gelaye Y. (2024). Application of precision agriculture technologies for sustainable crop production and environmental sustainability: a systematic review. Sci. World J. 2024, 1–12. doi: 10.1155/2024/2126734, 39421732 PMC11483651

[ref13] Goldstein M. Uchida S. (2016). A comparative evaluation of unsupervised anomaly detection algorithms for multivariate data. PLoS One 11:e0152173. doi: 10.1371/journal.pone.0152173, 27093601 PMC4836738

[ref14] Indiveri G. Liu S.-C. (2015). Memory and information processing in neuromorphic systems. Proc. IEEE 103, 1379–1397. doi: 10.1109/JPROC.2015.2444094

[ref15] Kim Y. Evans R. G. Iversen W. M. (2008). Remote sensing and control of an irrigation system using a distributed wireless sensor network. IEEE Trans. Instrum. Meas. 57, 1379–1387. doi: 10.1109/TIM.2008.917198, 25079929

[ref16] Lane N.D. Bhattacharya S. Georgiev P. Forlivesi C. Jiao L. Qendro L. (2016) DeepX: a software accelerator for low-power deep learning inference on mobile devices. In: 2016 15th ACM/IEEE International Conference on Information Processing in Sensor Networks (IPSN)

[ref17] Mehonic A. Sebastian A. Rajendran B. Simeone O. Vasilaki E. Kenyon A. J. (2020). Memristors—from in-memory computing, deep learning acceleration, and spiking neural networks to the future of neuromorphic and bio-inspired computing. Adv. Intell. Syst. 2:2000085. doi: 10.1002/aisy.202000085

[ref18] Neftci E. O. Mostafa H. Zenke F. (2019). Surrogate gradient learning in spiking neural networks. IEEE Signal Process. Mag. 36, 51–63. doi: 10.1109/MSP.2019.2931595

[ref19] Nunes J. D. Carvalho M. Carneiro D. Cardoso J. S. (2022). Spiking neural networks: a survey. IEEE Access 10, 60738–60764. doi: 10.1109/ACCESS.2022.3179968

[ref20] Pfeiffer M. Pfeil T. (2018). Deep learning with spiking neurons: opportunities and challenges. Front. Neurosci. 12:774. doi: 10.3389/fnins.2018.00774, 30410432 PMC6209684

[ref21] Rathi N. Srinivasan G. Panda P. Roy K. (2020). Enabling deep spiking neural networks with hybrid conversion and spike timing dependent backpropagation. arXiv.

[ref22] Shrestha A. Fang H. Mei Z. Rider D. P. Wu Q. Qiu Q. (2022). A survey on neuromorphic computing: models and hardware. IEEE Circuits Syst. Mag. 22, 6–35. doi: 10.1109/MCAS.2022.3166331

[ref23] Tace Y. Tabaa M. Elfilali S. Leghris C. Bensag H. Renault E. (2022). Smart irrigation system based on IoT and machine learning. Energy Rep. 8, 1025–1036. doi: 10.1016/j.egyr.2022.07.088

[ref24] Tavanaei A. Ghodrati M. Kheradpisheh S. R. Masquelier T. Maida A. (2019). Deep learning in spiking neural networks. Neural Netw. 111, 47–63. doi: 10.1016/j.neunet.2018.12.002, 30682710

[ref25] Venkatesha Y. Kim Y. Tassiulas L. Panda P. (2021). Federated learning with spiking neural networks. IEEE Trans. Signal Process. 69, 6183–6194. doi: 10.1109/TSP.2021.3121632

[ref26] Wang H. Li Y.-F. Gryllias K. (2024). Brain-inspired spiking neural networks for industrial fault diagnosis: a survey, challenges, and opportunities. arXiv.

[ref27] Xie K. Zhang Z. Li B. Kang J. Niyato D. Xie S. . (2022). Efficient federated learning with spike neural networks for traffic sign recognition. IEEE Trans. Veh. Technol. 71, 9980–9992. doi: 10.1109/TVT.2022.3178808, 25079929

